# The Clinique Médicale; or Reports of Medical Cases

**Published:** 1836-01

**Authors:** 


Art. V.—*The Clinique Médicale; or, Reports of Medical Cases:* by G. Andral. *Condensed and translated, with Observations extracted from the Writings of the most distinguished Medical Authors.* By D. Spillan, m.d. Part I.-II.-III. pp. 608. *London*, 1835. 8vo.

It will not be expected from us, after the lapse of a dozen years since the publication of the greater part of the work of Andral, that we should now enter upon any review or criticism of it. The work, in fact, has long since assumed the station in medical literature and in the estimation of all intelligent members of the profession, which it is for ever destined to occupy. "The merits of the original work," (to use the words of the advertisement to the present translation,) "are so universally acknowledged, and its character is so well established, as forming an era in the history of medical pathology, that it is altogether unnecessary to say anything regarding it in the way of eulogy. It contains a series of select cases, and observations eminently suited to illustrate the history and nature of those diseases which are of most frequent occurrence in the department of *internal pathology*. The manner in which these cases are arranged, and the mode in which the facts furnished by them are generalised, entitle it to rank as a *complete system of clinical instruction*."

Entertaining opinions scarcely less strong of the great merits of this work, it was with sincere pleasure that we saw a translation of it announced; and although, after examination, we cannot give unqualified praise to the English version now before us, either as a literary performance, or as a rigidly accurate transcript of the original, we are bound to acknowledge that it constitutes an important addition to the stock of English medical literature, and that the student in particular, who may be ignorant of the language in which it was written, or to whom the expense and bulk of the original were obstacles to its possession, is under great obligations to Dr. Spillan for the valuable and acceptable offering which he has here presented to him.

The external or mechanical qualities of the English version are most exemplary. The paper is remarkably good, the print—although somewhat of the smallest to delight the eyes of old critics, singularly clear and beautiful; the whole aspect of the work agreeable and elegant, and last, not least, the volume, its immense mass of matter being considered, is marvellously cheap. We therefore earnestly recommend all our young friends, who wish to know what is most excellent in the modern pathology of France, to lose no time in adding the Translation of the Clinique of Andral to their stock of medical authors and authorities. And we do this advisedly: we have already hinted our want of entire satisfaction with the work of Dr. Spillan, but its blemishes are rarely such as are likely to mislead the student, while it must be regarded, on the whole, as giving a faithful transcript of all the facts and inferences of the author. The faults of his translation are indeed more of a literary than professional kind, although occasionally he has sinned even against scientific fidelity.

Regarded as a whole the translation certainly cannot be called even very good; it has no pretensions to elegance; it savours too much by far of the "done into English" of the olden days of translation. It is by far too literal, and consequently, even in its new style, still too *French*. It is impossible to read a page—nay, hardly a paragraph—without knowing that you are reading a translation, and even a French translation; the awkward outlandish phrases constantly suggesting the well-known terms and phrases of the original. It is in vain that the Frenchman has been drilled into the correct employment of English words, his accent and the collocation of his words betray him at once; through the complete disguise of the national costume you detect the foreigner by the peculiarity of his gestures. This is the more to be lamented because the translator might certainly, with very little additional trouble, have eschewed the chief of these blemishes. If in place of at once and finally setting down the literal version of the words and of the exact turn of the style of the original, he had taken the trouble to consider for a moment whether they *sounded* or *looked* like genuine English; and where anything markedly foreign existed, had retranslated this into words having the same meaning, but a more native character, he would have produced a work much more creditable to himself and more agreeable to the reader of taste. We suspect the main source of his defects to be want of time; as we are certain, from the very good—nay, felicitous manner in which many passages are rendered, that he is very capable of giving a good translation of his author. He probably began his task without due consideration of its importance, or of the necessity for executing it with such a degree of precision as we conceive it ought to claim; and possibly the printer and publisher were more regardful of the value of time than of the translator's credit.

For all the inelegancies of the work now before us, the translator, however, is not responsible; unless, indeed, it be the duty of a translator to correct the inelegancies of his original. Andral is certainly a careless writer, and the present work, in particular, is full of repetitions, of sentences awkwardly arranged, and of phrases far from elegant, or even accurate, in a logical sense of the word. We conclude this notice with a few faulty passages which we have taken at random, as we dipped here and there into the work. When particularly struck with a turn of expression, or phrase, or word which seemed to us odd or wrong, we referred to the original, and generally found our suspicions of inelegance or inaccuracy confirmed.

Among the constantly recurring awkward expressions in the translation are the words "in fine" for the original "enfin." The word "precordial" very accurately and very properly means, both in the French and English,

"anterior to the heart;" but it is liable to be mistaken in our language, from the circumstance of *precordia* being almost constantly used in medical language, in the old sense of *epigastrium;* yet it is generally used in the other, or French sense, by Dr. Spillan. We might mention several other faulty expressions of frequent occurrence; but we proceed to notice a few out of the many blemishes which struck us in opening at random the pages of the Second Part, on the Diseases of the Chest.

P. 240. "*A great number of contractions* of the different orifices of the heart *recognise for their commencement* an acute or chronic inflammation," &c. This is certainly a literal translation of "Un grand nombre de rétrécissemens des differens orifices du cœur reconnaissent pour point du départ une inflammation," &c. but it is unquestionably not English.

P. 260. "But the blood, after escaping from its vessels, is merely accumulated and coagulated in this place, whilst at other times it was carried out according as it was deposited," &c. This is an incorrect version of the original "Mais seulement *en cet endroit* le sang, sorti de ses vaisseaux, s'est accumulé et coagulé, tandis *qu'ailleurs* il a été porté au dehors," &c. It is evident as well from the context as the obvious sense of the words, that *ailleurs* here applies to *other parts of the bronchi,* and ought to have been rendered *elsewhere,* with the verb in another tense.

P. 319. "Sputa *red,* transparent, viscid, still *detached* from the vessel by inclining it." This is a very bad translation of the original "Crachats *rouillés,* transparens, visqueuse, *se detachant* encore du vase lorsqu' on l'encline:" *rouillés* signifies *rusty-coloured,* or at most *reddish;* "still detached from" is, to make the best of it, a very awkward expression. In the same page we have the phrase "the blood drawn in the night was *formed by a clot,*" &c.; and this is one of the instances where the original author may be criticised as well as the translator, as the expression "le sang etait formé" seems not more accurate than its literal rendering in English. Still it must be admitted that such a mode of expression is common in France, but certainly not in England. Again in the same page we have the expression "he complained of a smothering," which, to say the least of it, is an inelegant rendering of the original phrase, "il se plaignait d'etouffer et invoquait la mort."

P. 360. "The intelligence again restored," although a literal translation of the original "l'intelligence avait reprit toute sa netteté," is not English; nor is "torrent of the circulation," "seroso-purulent;" "he could lie down in all positions," "tubercles, commencing to soften, existed," &c.; "the crepitation becomes so little," "pains which ran through the different articulations;" "respiratory tree," &c., and many others of a like kind that meet the eye in every page.

P. 286. "We found in fact," "nous retrouvons," should of course be in the present time, "we find." And in page 320, "S'il y avait eu hépatisation, un son mat aurait existé," is translated "if *there were hepatization,* the sound would have been dull:" it should be "if there had been."

These blemishes, taken individually, may appear slight; but when multiplied, they detract not a little from the merit of a work.

We trust the translator will receive our criticisms in good part, and will take more pains with any other work which he may think of transplanting from a foreign to his native soil. He ought to recollect that the great value of the materials does not justify a slovenly architecture, but, on the contrary, renders its defects more intolerable. No doubt, the intrinsic merits of Andral's work will render it a permanent favourite with the public; and we seriously counsel Dr. Spillan to go over his translation once more, and carefully, from beginning to end; and, having corrected the numerous defects and inelegancies which his own taste will point out to him, in despite of the obstacle of *Stereotype*, announced on his covers, reproduce, in a new edition, when the present is expended, a work more worthy of the great original, and more beseeming his own character as a scholar and physician.

